# Problematic Internet Users Show Impaired Inhibitory Control and Risk Taking with Losses: Evidence from Stop Signal and Mixed Gambles Tasks

**DOI:** 10.3389/fpsyg.2016.00370

**Published:** 2016-03-17

**Authors:** Qi Li, Weizhi Nan, Jamie Taxer, Weine Dai, Ya Zheng, Xun Liu

**Affiliations:** ^1^Key Laboratory of Behavioral Science, Institute of Psychology, Chinese Academy of SciencesBeijing, China; ^2^University of Chinese Academy of SciencesBeijing, China; ^3^Stanford Psychophysiology Laboratory, Department of Psychology, Stanford UniversityStanford, CA, USA; ^4^Department of Psychology, Dalian Medical UniversityDalian, China

**Keywords:** problematic Internet use, inhibition response, risk taking with losses, cognitive control, reward processing

## Abstract

According to the balance model of self-regulation, dysfunction of the inhibitory control and reward processing might be a behavioral marker for addiction and problematic behaviors. Although several studies have separately examined the inhibitory control or reward processing of individuals exhibiting problematic Internet use (PIU), no study has explored these two functions simultaneously to examine the potential imbalance of these functions. This study aimed to investigate whether the self-regulatory failure of PIU individuals results from deficits in both inhibitory control [indexed with the stop signal reaction time (SSRT) in a stop signal task] and risk taking with losses (measured as the acceptance rates of risky gables or the ratio of win/loss in a mixed gambles task). The results revealed that PIU individuals, compared with controls, showed decreased SSRT and increased error rates as well as reduced risk taking with losses. Correlational analyses revealed a significant positive relationship between the SSRT and risk taking with losses. These findings suggest that both the inhibitory control and reward functions are impaired in PIU individuals and reveal an association between these two systems. These results strengthen the balance model of self-regulation theory’s argument that deficits in inhibitory control and risk taking with losses may assist in identifying risk markers for early diagnosis, progression, and prediction of PIU.

## Introduction

The Internet plays a vital communication and social interaction role in modern life (Tonioni et al., 2012). However, some individuals are unable to control their Internet use, which can eventually cause serious mental health problems and a variety of negative psychosocial consequences (Ko et al., 2013b). This behavioral phenomenon is commonly referred to as problematic Internet use (PIU; Tsitsika et al., 2011). Although the description of PIU is based on the definition of substance dependence or pathological gambling, which are both examples of self-regulatory failure (Zhou et al., 2010), few studies have examined the self-regulatory failure of PIU.

Compared to the large number of individuals with online experience, why do only a few individuals become addicted? One possible explanation is that individuals who become addicted display deficits in self-regulation. According to the balance model of self-regulation, dysfunction of inhibitory control and reward processing might be a behavioral marker for addiction and problematic behaviors (Heatherton and Wagner, 2011). The balance model of self-regulation suggests that self-regulatory failure occurs as a result of a failure to appropriately engage top-down control mechanisms and bottom-up reward information. For instance, when self-regulatory resources are depleted (Gailliot et al., 2007; Muraven, 2010) or when impulse inhibition is impaired (Dong et al., 2010, 2012), people become prone to self-regulation failure in a top-down manner. Alternatively, when an individual is confronted with a strong impulse (e.g., an enticing dessert for someone on a diet), the likelihood of self-regulation failure in a bottom-up manner is increased. Thus, examining inhibitory control and reward processing in people with PIU might be an effective and useful way to understand their difficulty with self-regulation.

Several lines of research have identified an association between self-regulatory failure and impairments in inhibitory processes. For instance, several studies have found that self-reported impairments in control are reliably associated with greater past and future substance use (Gullo et al., 2014; Leeman et al., 2014a,b). Additionally, numerous studies using “go/no-go” or “stop signal” tasks provide converging evidence that individuals who are dependent on alcohol (Lawrence et al., 2009; Papachristou et al., 2013), cigarettes (Billieux et al., 2010), cocaine (Colzato et al., 2007), and food (Svaldi et al., 2014) display more difficulty inhibiting their responses than do controls, and deficits in behavioral response inhibition were found to be related to the severity of reported symptoms. Researchers have also observed inhibitory deficits in other addiction-like behavioral disorders that do not involve substance ingestion, namely, pathological gambling and PIU. For example, pathological gamblers exhibit performance deficits in go/no-go (Goudriaan et al., 2005; van Holst et al., 2012) and stop signal tasks (Goudriaan et al., 2006; Odlaug et al., 2011). Furthermore, a clinical study found that memantine treatment, which can reduce glutamate excitability and improve impulsive decision making, is associated with diminished gambling and improved cognitive flexibility (Grant et al., 2010). The link between inhibitory deficits and PIU have so far been mixed. Some studies have found that in comparison to controls, PIU individuals exhibit inhibitory deficits in the go/no-go task (Dong et al., 2010; Zhou et al., 2010; Liu et al., 2014) and stop signal task (Choi et al., 2013, 2014); in contrast, one study reported that PIU individuals performed better in the go/no-go task than controls (Sun et al., 2009). Notably, the reaction stimuli in this task are always go targets and no-go non-targets; therefore, the differences in these stimuli may reflect only aspects of the target (go)/non-target (no-go) decisions rather than the active suppression of motor responses. Given the limited amount of research, more research on inhibitory deficits in PIU individuals is warranted.

In addition to inhibitory control, exploring the relationship between self-regulatory failure and dysfunction of reward processing could have significant implications for research and treatment. Most research on addiction disorders has primarily focused on reward anticipation and reward outcome processing during gain and loss conditions; such studies have revealed that individuals with alcohol dependence (Wrase et al., 2007; Beck et al., 2009), cigarette smoking behavior (Rose et al., 2013), gambling problems (Dong et al., 2011; Ko et al., 2013a; Bustamante et al., 2014), and Internet addiction (Dong et al., 2011; Ko et al., 2013a,b) exhibit deviant reward processing. However, ambiguous findings have been reported for cocaine addiction (Jia et al., 2011; Bustamante et al., 2014). For example, some studies have observed increased anticipatory gain activity in a treatment-seeking group with cocaine dependence (Jia et al., 2011), whereas other studies have reported diminished anticipatory gain processing in cocaine-dependent patients with 1–2 years of sobriety (Bustamante et al., 2014). In fact, in addition to the clinical differences, which include treatment status, length of abstinence, drugs or drug metabolites, and other comorbidities, the varying sensitivity to gains and losses might be the key to explaining the variability among these findings.

Thus, risk taking with losses could also be a critical aspect of reward processing that provides vital insights into the self-regulatory failure of PIU and other behavioral disorders. When faced with a risky decision, individuals are often considerably more concerned with the potential loss rather than with the potential gain of the same magnitude. We are referring to this behavioral phenomenon as risk taking with losses. This phenomenon may result from an asymmetric sensitivity in reward responses in which losses “loom larger” than gains (Kahneman and Tversky, 1979). Risk taking with losses is associated with the “decision” utility of potential losses versus gains when a risky decision is being made (Tom et al., 2007). High risk taking with losses is an indication of the potential gain being more dominant for an individual than the potential loss. High risk taking with losses could reflect a decreased sensitivity to losses due to individual differences in reward processing (Tremeau et al., 2008; Lorains et al., 2014). A recent study demonstrated that problem gamblers took higher risks with losses than controls in a mixed gambles task (Lorains et al., 2014). Using the same task, another study found that pathological gamblers in earlier stages of therapy took higher risks with losses and accepted a higher number of gambles than did pathological gamblers in later stages of therapy; hence, sensitivity to risk taking with losses increased as a result of clinical treatment (Giorgetta et al., 2014). However, to our knowledge, no research has investigated the potential outcomes of risk taking with losses, a fundamental aspect of reward processing, in PIU individuals and those with substance addictions.

In the present study, we examined the inhibition control and risk taking with losses of PIU individuals in comparison to non-PIU individuals. As a measure of inhibition in substance use problems, the stop signal task has been found to be more sensitive to deficits than the go/no-go task (Smith et al., 2014), therefore, we used the stop signal task to capture the inhibitory deficits of PIU individuals. In general, performance in the stop signal task has been effectively modeled using the horse-race model (Band et al., 2003). This model assumes that stop (inhibitory) and go (executed) processes operate independently and that the response is stopped or executed depending on which set of processes wins the race (Kok et al., 2004). Hence, participants are more likely to fail at inhibiting their responses when the execute process finishes first. Moreover, to investigate risk taking with losses in PIU individuals, a mixed gambles task was used to investigate individual’s reactions to potential losses versus gains during decision making (Tom et al., 2007). Based on the balance model of self-regulation, we hypothesized that PIU individuals would exhibit impaired functioning in inhibition control and/or risk taking with losses.

## Materials and Methods

### Participants and Procedure

Right-handed participants were recruited via advertising posted on the Bulletin Board System and on campus. An experienced psychiatrist screened potential participants with the Structured Clinical Interview for DSM-IV, which excludes the axis I psychiatric disorders. The Young Diagnostic Questionnaire for Internet Addiction (YDQ; Young, 1998) was used to identify PIU individuals, 32 respondents who answered “yes” to at least five questions were classified as suffering from PIU (18 males; aged 18–24 years, *M*_age_ = 21.13, *SD*_age_ = 1.60; years of education: *M* = 15.84, *SD* = 1.44), and 34 age-, gender-, and education-matched healthy individuals with YDQ scores of less than or equal to four were selected as the control group (20 males; aged 18–24 years, *M*_age_ = 20.97, *SD*_age_ = 1.64; years of education: *M* = 15.84, *SD* = 1.44 years). Prior to the study, all participants voluntarily enrolled in the study and signed an informed consent statement in accordance with the Declaration of Helsinki. The study was approved by the Institutional Review Board of the Institute of Psychology of the Chinese Academy of Sciences. All participants completed the “stop signal” task and the “mixed gambles” task, with the order of the tasks counterbalanced across participants.

### Behavioral Tasks

#### Stop Signal Task

A version of the stop signal task was administered to study the behavioral inhibition of PIU individuals. This task consisted of one deadline estimation block with 50 trials, one training block with 16 trials, and six testing blocks with 256 trials. During the deadline estimation block, the participants were asked to perform a letter discrimination task. Half of the participants in each group (control and PIU) were asked to press the “F” key with their left index finger in response to the letter “A” and to press the “J” key with their right index finger in response to the letter “B.” The other half of the participants was trained to perform the opposite pairing. Each trial began with a fixation point in the center of the screen for 100 ms followed by a letter inside a green box for 1200 ms. The trial ended with a fixation point of 1500 ms.

**FIGURE 1:**
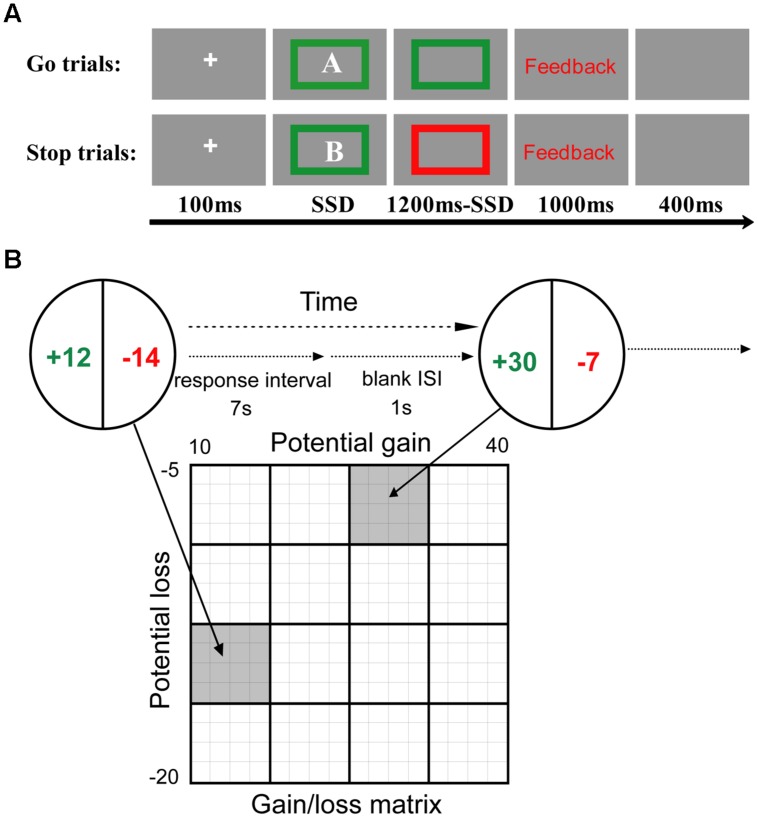
**Behavioral tasks. (A)** The stop signal task. In this task, the participants were asked to discriminate between the letters “A” and “B.” In 33.3% of the trials, the “stop” signal, a red box surrounding the letter (opposed to the box remaining green in the “go” trials), was presented at variable delays after the letter was displayed. The total duration of the onscreen display of the letter and color box was 1200 ms. The time interval between the go and stop signals (the stop signal delay, SSD), began at 300 ms and was updated with a step of 50 ms per trial using a staircase procedure. **(B)** The mixed gambles paradigm. The participants were asked to accept or reject mixed gambles offering equal (50%) chances of gaining or losing different amounts of money. All combinations of gains and losses were presented. ISI, interstimulus interval.

During the training and testing blocks, the participants continued to complete the letter discrimination task (**Figure 1A**); however, they were informed that some “stop” trials were added to the current task. That is, in the “go” trials participants continued completing the letter discrimination task as they had done in the deadline estimation block. In the “stop” trials, a “stop” signal (the green box surrounding the letter turned red) indicating that participants should withhold their response to the letter discrimination task was presented at variable delays after the letter was displayed. The “go” and “stop” trials were randomly intermixed, with “stop” trials constituting one-third of all trials. To ensure that participants would not improve their accuracy by reducing their speed, participants were told that slow responses would be regarded as “wrong.” “Slow” responses were determined by estimating the 90th percentile of an individual’s reaction time (RT) in the letter discrimination task during the deadline estimation block.

The trials in the training and testing blocks each began with a fixation of 100 ms followed by a letter in a colored box for 1200 ms, during which participants were allowed to respond up to a pre-estimated deadline from the onset of the letter. The response was followed by feedback (right, wrong, or slow) for 1000 ms. The trial ended with a 400 ms blank screen. The time interval between the go and stop signals, the stop signal delay (SSD), was initially 300 ms and varied from one stop trial to the next according to a staircase procedure. After a successful stop trial, the SSD was increased by 50 ms, and after a failed stop trial, it was decreased by 50 ms, thereby converging at a “critical” SSD and resulting in an approximately 50% successful inhibition rate (Levitt, 1971).

#### “Mixed Gambles” Task

A version of a “mixed gambles” task was used to study PIU individuals risk taking with losses. This task included four blocks. Prior to the “mixed gambles” task, all participants were given ¥50 for their participation in the questionnaire and the stop signal task. To convince the participants that they would be engaging in a real gambling task, they were told that one decision from each of the four blocks would be honored with real money, and an additional ¥50 was given for the present task.

In the mixed gambles task, the participants were offered a 50/50 chance of gaining one amount of money or losing another amount (**Figure 1B**). Possible gains ranged from ¥10 to ¥40 (in ¥2 increments), and possible losses ranged from ¥5 to ¥20 (in ¥1 increments), and these amounts were manipulated independently to allow for separate estimates of responses to gains and losses. All 256 possible combinations of gains and losses were presented randomly across the four blocks. The participants were asked to decide whether to accept or reject each of the gambles presented to them. If they accepted a gamble, the outcome was decided with a coin toss; if they rejected a gamble, then the gamble was not played. To encourage the participants to reflect on the subjective attractiveness of each gamble rather than to comply with a fixed decision rule (e.g., accept the gamble only if gain ≥ 2 × loss), they were given 6 s to indicate the extent to which they accepted or rejected (i.e., strongly accept, weakly accept, weakly reject, and strongly reject) each gamble. This was done by pressing the S or D key with their left ring or middle finger to indicate that they either “strongly accept” or “weakly accept,” or by pressing the K or L keys with their right middle or ring finger to indicate they either “weakly reject” or “strongly reject.” When a participant made a choice, the chosen option was then highlighted by a thick red outline around the chosen rectangle for 1 s. The alternatives then disappeared, and a blank showed for 1 s. Due to the positive expected value of the gambles that the participants evaluated, no participant actually lost from these gambles. The average amount won from gambling was ¥15 (max gain = ¥24 and min gain = ¥4). Given the initial ¥50 endowment, all participants finished this task with a net gain ranging from ¥54 to ¥74.

### Measures

After the experiment, the participants were asked to complete Chinese translations of the Barratt Impulsiveness Scale-11 (BIS-11; Patton et al., 1995) and the Behavioral Inhibition System/Behavioral Activation System questionnaire (BIS/BAS; Carver and White, 1994). The BIS-11 is a 30-item questionnaire assessing impulsiveness on a scale from 1 (rarely/never) to 4 (almost always/always). The BIS-11 includes three impulsiveness subscales: attentional, motor, and non-planning. The overall impulsiveness score is determined by summing the items from the three subscales, with higher scores indicating greater impulsivity. The BIS/BAS questionnaire has been widely used to assess individual differences in two motivational systems, the aversive and appetitive systems. The questionnaire is composed of 20 items that are divided into two primary scales: the BIS (seven items) and the BAS (13 items). The BAS scale includes three subscales: Reward Responsiveness (BAS-Reward; five items), Drive (BAS-Drive; four items), and Fun Seeking (BAS-Fun; four items). Each item is answered using a four-point Likert scale ranging from 1 (strongly disagree) to 4 (strongly agree). **Table 1** shows the demographic characteristics, descriptive statistics and group differences of the PIU and control participants on the BIS-11 and BIS/BAS.

**Table 1:** Demographic information, means (and standard deviations), and group differences between Individuals with PIU and controls.

	Individuals with PIU	CON	*p*-value
Age (years)	21.13 (1.60)	20.97 (1.64)	0.7
Gender (M/F)	18/14	20/15	0.94
Education (years)	15.84 (1.44)	15.57 (1.36)	0.43
YDQ	5.69 (1.47)	1.71 (1.30)	<0.001
BIS-11	72.63 (13.41)	58.49 (11.19)	<0.001
Attentional	19.59 (3.94)	15.11 (3.64)	<0.001
Motor	24.19 (5.15)	19.51 (4.13)	<0.001
Non-planning	28.84 (5.73)	23.85 (4.55)	<0.001
BIS	21.16 (2.00)	20 (3.00)	>0.05
BAS	45.91 (4.55)	43.97 (3.20)	<0.05
BAS-Reward	18.72 (1.71)	18.06 (1.64)	>0.05
BAS-Drive	13.63 (2.08)	13.89 (1.49)	>0.05
BAS-Fun	13.56 (2.05)	12.03 (1.92)	<0.01

*Mean group differences were examined with a two-sample *t*-test and a chi-square test was used for gender differences between groups. PIU, problematic Internet use; CON, controls; YDQ, Young’s Diagnostic Questionnaire for Internet Addiction; BIS-11, Barratt Impulsiveness Scale-11; BIS, Behavioral Inhibition System Scale; BAS, Behavioral Activation System Scale*.

### Data Analysis

#### “Stop Signal” Task

Based on the horse-race model which asserts that the go and stop processes compete with one another in their race toward the finish line (Logan, 1994), the stop signal reaction time (SSRT) was computed by subtracting the critical SSD from the median RT in go trials. A longer SSRT indicates poor response inhibition. In the current stop signal task, an independent-samples *t*-test was used to compare the SSRT, RT in go trials, and percentage of errors in go trials of the PIU and control groups. Pearson correlation coefficients were used to examine the interrelatedness of SSRT and both rate of PIU and impulsivity.

#### “Mixed Gambles” Task

Statistical analyses were performed with MATLAB R2009b (http://www.mathworks.com). As a first step, the strong/weak responses of each participant were transformed into accept and reject categories. Next the acceptance rates of risky gambles (*P*) were computed. Then, a logistic regression was ran with the sizes of the potential gain and loss entered as independent variables and accept and reject categories entered as dependent variables. The risk taking with losses (λ) was computed as follows: λ = –β_loss_/β_gain_, where β_loss_ and β_gain_ are the unstandardized regression coefficients for the loss and gain variables, respectively (Tom et al., 2007). In the current mixed gambles task, the acceptance rates (*P*) and the size of log (λ) were compared between the PIU and control groups using an independent-samples *t*-tests.

Pearson correlation coefficients were utilized to examine the relationships between the participants acceptance rates (*P*) as well as their risk taking with losses log (λ) and their YDQ scores and SSRT. The alpha level was set at 0.05 for all analyses.

## Results

### “Stop Signal” Task

**FIGURE 2:**
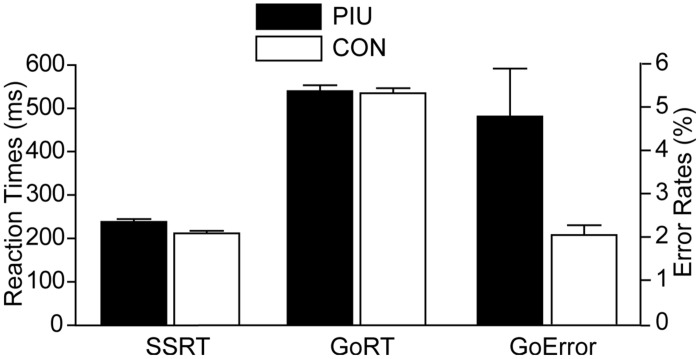
**Stop signal task performance group means and 95% confidence intervals for three measures: stop signal reaction time (SSRT; an index of response inhibition), reaction time in go trials (GoRT), and error rates in go trials (GoError)**. SSRTs and RTs were in ms (left *y*-axis), whereas error rates were in percentages (right *y*-axis). PIU individuals had significantly longer SSRTs and higher error rates than the control group, but they did not significantly differ in RTs measures.

The success rates of inhibition in the stop signal trials were 49.88% for the PIU group and 50.99% for the control group; the lack of group differences, *t*(65) = 1.13, *p* > 0.05, indicates that the current procedure was successful. The PIU group (*M* ± *SD* = 238 ± 37 ms) had slower SSRTs, *t*(65) = –3.05, *p* < 0.01, and higher error rates in go trials, *t*(65) = 2.54, *p* < 0.05, than the control group (*M* ±*SD* = 212 ± 32 ms). However, the groups did not significantly differ in their go trial RTs, *t*(65) = –0.42, *p* > 0.05 (**Figure 2**). Furthermore, the SSRTs of all participants were significantly correlated with their YDQ scores (*r* = 0.32, *p* < 0.01), as well as total BIS-11 scores (*r* = 0.46, *p* < 0.001) and its three subscales (attention: *r* = 0.40, *p* < 0.01; motor: *r* = 0.43, *p* < 0.001; non-planning: *r* = 0.45, *p* < 0.001). The YDQ scores were positively correlated with the total BIS-11 scores (*r* = 0.63, *p* < 0.001) and all three subscales (attention: *r* = 0.66, *p* < 0.001; motor: *r* = 0.54, *p* < 0.001; non-planning: *r* = 0.56, *p* < 0.001).

### “Mixed Gambles” Task

**FIGURE 3:**
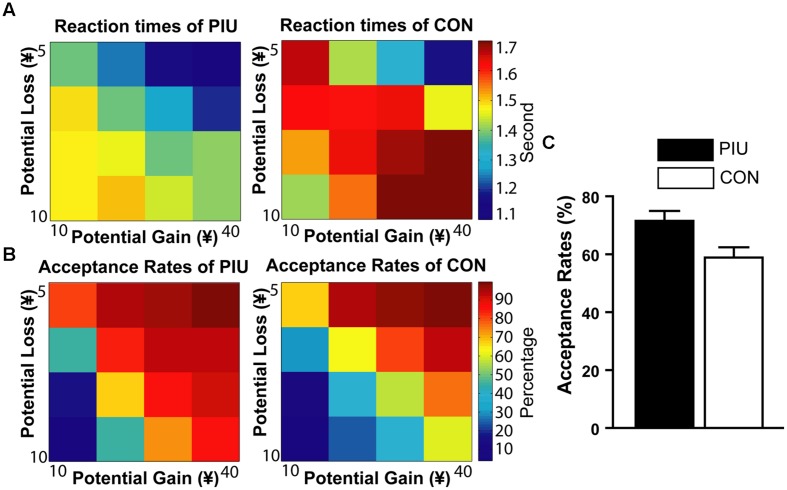
**Mixed gambles task performance at each level of gain/loss. (A)** Color-coded heatmap of RTs for the PIU and control groups (red indicates slower RTs and blue indicates faster response times). **(B)** Color-coded heatmap of the percentage of gamble acceptance for the PIU and the control groups (red indicates a strong willingness to accept the gamble, and blue indicates a low willingness to accept the gamble). **(C)** PIU individuals had significantly higher acceptance rates of risky gables (*P*) than the control group.

The acceptance rates (*P*) of the PIU group (*M* ±*SD* = 71.54% 19.28%) were significantly larger than that of the control group (*M* ±*SD* = 58.60%, ± 20.63%, *t*(65) = 2.65, *p* < 0.01). The control group’s ratio of loss responses to their gain responses or their risk taking with losses λ (*M* ±*SD* = 2.27 ± 1.10) was consistent with previous findings (Tom et al., 2007). That is, similar to the difference observed for gambles in which the potential gain was twice the amount of the potential loss, the control group was slower and more hesitant in deciding whether to accept the gambles. However, the risk taking with losses λ (*M* ±*SD* = 1.54 ± 0.51) of the PIU group was significantly smaller than that of the control group, *t*(65) = 4.02, *p* < 0.001 (**Figure 3**).

**FIGURE 4:**
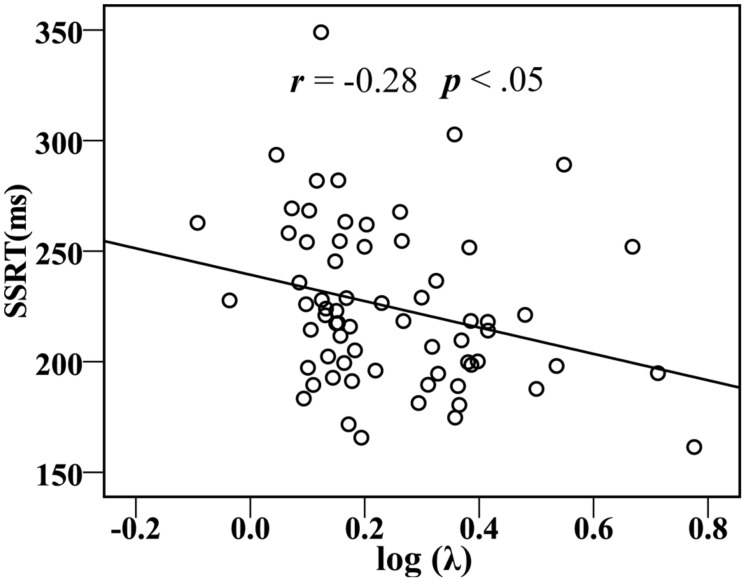
**Correlations between the SSRT and behavioral or the ratio of win/loss in risky gables (log λ)**.

In addition, for all participants, acceptance rates (*P*) were marginally significantly correlated with YDQ scores (*r* = 0.23, *p* = 0.059), and the risk taking with losses log (λ) was significantly correlated with YDQ scores (*r* = –0.33, *p* < 0.01), the Fun-Seeking subscale of the BAS (BAS-Fun: *r* = –0.32, *p* < 0.01), and the SSRTs (*r* = –0.28, *p* < 0.05; **Figure 4**).

## Discussion

This study was the first to simultaneously examine inhibitory control and reward processing in PIU individuals using a stop signal task and a mixed gambles task, respectively. Our study showed that PIU individuals, compared with controls, displayed an inability to inhibit responses and a diminished sensitivity to risk taking with losses. Moreover, the more individuals were able to inhibit their responses (shown through the SSRT), the lower their behavioral risk taking with losses was. Together, these results strengthen the argument that deficits in inhibitory control and risk taking with losses may offer promising opportunities to identify the underlying of excessive Internet use observed in PIU individuals. The present study indicates that PIU individuals might have more difficulty controlling their Internet use and be less sensitive to the negative consequences involved in excessive Internet use, and therefore, continue their behavior despite adverse consequences.

Using the stop signal task, which is an effective method to manipulate response inhibition, the present study identified the deficits in inhibitory and executed responses that underlie self-control in individuals with PIU. As previously mentioned, the horse-race model, which assumes that stop (inhibit) and go (execute) processes are in competition with one another, provides a quantitative interpretation of performance in the stop signal task (Band et al., 2003; Kok et al., 2004). In the present study, the time for stopping the response was estimated with the SSRT. We found that in comparison to controls, PIU individuals required more time to inhibit responses. Notably, the slower response inhibition among PIU individuals could not be attributed to in general faster response execution of controls, because both groups displayed similar mean RTs in go trials. The slower response inhibition could be the result of a general slowing of the inhibitory process, such that more time is required to inhibit a response. Furthermore, although PIU individuals did not differ from controls in their ability to execute responses (reflected in go-trial RTs), PIU individuals did display higher error rates in go trials, which demonstrates an impaired execution response in PIU individuals. This finding is consistent with previous research (Zhou et al., 2014). Previous studies using variants of the go/no-go task have also found that individuals with severe PIU exhibit deficient inhibitory control, and this deficiency was confirmed by behavioral, electro-physiological brain potential and functional brain imaging (Dong et al., 2010; Zhou et al., 2010).

The present findings from the mixed gambles task are the first to directly compare quantifiable measures of risk taking with losses between PIU individuals and controls. We found that in comparison to controls, individuals with PIU decided more frequently to accept gambles during the mixed gambles task. Compared with controls, PIU individuals tended to exhibit higher behavioral risk taking with losses, and required less time to decide whether to accept gambles. These findings provide evidence of a specific increase in risk taking with losses in PIU individuals — a finding that is consistent with previous research which found that PIU individuals have enhanced reward sensitivity and decreased loss sensitivity compared to controls during a guessing task (Dong et al., 2011).

The exact reason for the increase in risk taking with losses in PIU individuals is still unclear. One possibility is that individuals with PIU had a diminished sensitivity to the potential loss. According to the regulatory focus theory (Higgins, 1997), people are usually guided by two distinct motivational systems. One involves a promotion focus for potential positive rewards (gains), such as advancements, growth, and accomplishments, and the other involves a prevention focus for potential negative rewards (losses), such as protection and safety. Accordingly, it seems that individuals with PIU have a strong system for promoting potential positive rewards and a lower system for preventing potentially adverse consequences (Dong et al., 2011). Within the present study, when individuals with PIU were confronted with a risky decision, they did not generate strongly negative reward responses, and they predominantly neglected to consider the negative aspect of the risk, and thus, they preferred adventure by exhibiting higher tendencies toward risk taking with losses. Another possibility is that non-PIU individuals were avoiding delay. Only accepting the gamble was associated with a delay (the coin toss), while rejecting it was associated with no delay. Thus, a decrease in risk taking with losses may simply reflect wanting to be done with the experiment faster (Silberberg et al., 2008). The third possibility is that PIU individuals are more likely to take risks (independently of gains or losses). The present study did not include a control task without losses and therefore PIU individuals may simply have taken more risk in the sense of preferring variance over a fixed outcome (Yechiam and Hochman, 2013).

In addition, this study provided empirical evidence of the relationship between inhibitory control and reward processing in PIU individuals. Many previous studies have found a dysfunction in either the inhibitory control or reward processing of individuals with substance dependence (Parvaz et al., 2012; Kamarajan et al., 2013), problem gambling (Goudriaan et al., 2005; Lorains et al., 2014), and PIU (Dong et al., 2010, 2011), and such findings have supported the balance model of self-regulation. That is, dysfunction of inhibitory control or reward processing might be a behavioral marker for addiction or other behavioral disorders (Heatherton and Wagner, 2011). However, the neurobiological model of adolescent development proposes that top-down control and bottom-up reward systems should be considered together (Casey et al., 2008). The combination of heightened responsiveness to rewards and immaturity in behavioral control may bias adolescents toward seeking immediate gains rather than focusing on long-term losses, perhaps explaining their increased tendency to engage in various addictive and risky behaviors. Conforming to this model, the PIU individuals in our study demonstrated less effective inhibitory behavior and more excessive fun seeking. Furthermore, we found that individuals who took longer to inhibit their responses, tended to be less aversive to losses. This result suggests that impairment of the functions of inhibitory control and reward processing in PIU individuals is not independent but linked. Dysfunctions in both of these systems might be markers of risk for PIU and various risk behaviors.

The present study also included some limitations. This was a cross-sectional study so even though in comparison to controls, PIU individuals showed a dysfunction of inhibitory control and reward processing, it is hard to determine whether the dysfunction of these features preceded the development of PIU or were a consequence of the overuse of the Internet. Therefore, further studies should tease apart the causal relations between PIU and these features. Secondly, the sample size in this study was relatively small, which might reduce the power of the statistical significance and generalization of the findings. Owing to this limitation, these results should to be considered preliminary and need to be replicated in future studies with a larger sample size.

## Conclusion

The present study revealed that inhibitory control and reward processing were simultaneously impaired in PIU individuals. Importantly, the present study illustrated an association between these two systems that suggested an imbalance of self-regulation in PIU individuals as a result of the diminished function of both systems. Moreover, the present results for PIU individuals may provide insight into a number of neuropsychiatric and behavioral disorders associated with self-regulatory failure, such as substance abuse, pathological gambling, and antisocial personality disorder. However, future studies should integrate methods related to both inhibition response and reward processing to gain greater insight into the mechanisms underlying the development of PIU. To promote the development of specific prevention and treatment procedures, further longitudinal research revealing the causes and consequences of PIU is needed to explore the role of inhibitory control and sensitivity to rewards in predicting the development of PIU.

## Author Contributions

QL designed the experiments and wrote the paper. JT and YZ edited this manuscript. WN and WD conducted the experiments and analyzed these data. XL designed the experiments and gave some suggestions to edit this manuscript.

## Conflict of Interest Statement

The authors declare that the research was conducted in the absence of any commercial or financial relationships that could be construed as a potential conflict of interest.
